# A New Method for Operating upon Loose or Sensitive Teeth

**Published:** 1855-01

**Authors:** W. A. Helme

**Affiliations:** Dentist, Providence, R. I.


### ARTICLE III.

### *A New Method for Operating upon Loose or Sensitive Teeth.*

By W. A. HELME, Dentist, Providence, R. I.

I HAVE recently adopted a method which enables me to operate on loosened, sensitive teeth, in some of the forms of disease frequently seen; and with such favorable results that I send you a report of the following cases as descriptive of it, and hope it may be of interest to yourselves and to the readers of the *Journal.*

A lady called on me for advice concerning a *dead* superior incisor, which was protruded from its socket, loose and painful from excessive inflammation of the periosteum. The constant exposure to violent contact with the lower teeth, consequent on its protrusion, allowed no opportunity for the inflammation to subside. It was also unsightly, being a line and a half longer than its fellow.

The obvious indication was to shorten the tooth, but this seemed quite impossible from the extreme pain occasioned by the slightest touch, even of the tongue. It was while considering this case that the plan occurred to me by which the operation might be made without further loosening the tooth or causing pain.

Having obtained the consent of my patient to the proposed experiment, I prepared a mixture of plaster of paris and moulded it with a palate-knife about all the front teeth, enclosing the incisors and cuspidati in such a manner that it left only their cutting edges exposed. In a few moments the plaster hardened sufficiently, and the diseased tooth was held in its place with a vice-like firmness, and upon trial, I found it was no longer sensitive to pressure, or to the jarring sensation of the file, with which I cut it down, though I used a much coarser one than I had ever before considered expedient to use upon teeth.

The operation gave the patient *no pain or annoyance whatever*, on the contrary, during the whole time of the operation she was laughing at the ridiculous figure she fancied she made, with the ball of plaster on her teeth. When the plaster was removed, she said no words could express how entirely she was satisfied with the result of the experiment.

The success of this case, brought, on the following day, a gentleman, a brother of the lady, with a loose lateral incisor, which had so much decayed that it seemed impossible to fill it without breaking or starting from its socket, in which it was but slightly retained. However, I resorted to my new plan of support, and backed up the tooth in such a manner that it left the decayed side fully exposed, and yet so surrounded, that it was held in its position with great firmness as the plaster hardened. I then proceeded to excavate and fill the cavity being able to condense the plug with impunity, the great pressure being distributed by this means all along that portion of the dental arch enclosed by the plaster. The satisfaction of the patient was great, as he had by previous advice abandoned all hope of saving his tooth.

Again I filled two central incisors which had been separated two days before by gum elastic and were consequently inflamed and sensitive. The first was supported as well as possible with the thumb and fingers in the usual way, but the operation gave pain. The second tooth appearing to be still more sensitive, I tried the plan of supporting it with plaster and successfully as in this instance there was *no pain.*

Since that time, I have resorted to the above mode of treatment for similar cases, both in the upper and lower jaw, and always with success, so that I now regard the use of plaster as essential in my practice in treating teeth under such circumstances, and believe any dentist who will give the plan a fair trial will adopt it, in cases where pain may be avoided by a firm support.

The plaster should be of the best quality, and mixed much thicker than for ordinary use in dental manipulations, and allowed to stand until it is nearly ready to *set* before using. Apply it with a small spatula, moulding it about the teeth and gums, both within and without the arch, in sufficient quantity to afford the requisite strength. A little practice will determine how much may be necessary for any given case—perhaps the whole mass should generally be about three-fourths of an inch in thickness, or more for long operations. It is better to apply too much than too little, for if too little is used, it may, with the best care, absorb sufficient saliva to soften it before the operation is concluded; especially when applied to lower teeth. In my first trial I made a mistake in this respect and was obliged to repeat the application.

To remove the plaster it is only necessary to first saturate it well with water.

The plaster being perfectly clean and tasteless, there is no unpleasant effect in using it in the mouth in this way, and we trust that any fear arising from its *seeming* unfitness will not prevent the use of what may save so much pain.

The skillful dentist, while he performs his duty with an unflinching hand, should resort to every expedient to alleviate the sufferings which he is so often called upon to inflict.

